# Novel insights into how gestational diet affects maternal-infant microbiota: a cross-sectional causal mediation analysis at one month postpartum

**DOI:** 10.1007/s00394-026-03909-9

**Published:** 2026-03-09

**Authors:** Eduard Flores Ventura, Sergio Ruiz-Saavedra, Raúl Cabrera-Rubio, Cecilia Martínez-Costa, Sonia González, Maria Carmen Collado

**Affiliations:** 1 Department of Biotechnology, Institute of Agrochemistry and Food Technology-National Research Council (IATA-CSIC), Agustin Escardino 7, 46980 Paterna, Valencia, Spain; 2 PhD Program in Biotechnology, Universitat Politècnica de València, 46022, Camí de Vera s/n, Valencia, Spain; 3 Diet, Microbiota and Health Group, Instituto de Investigación Sanitaria del Principado de Asturias (ISPA), 33011 Oviedo, Spain; 4 Department of Microbiology and Biochemistry of Dairy Products, Instituto de Productos Lácteos de Asturias (IPLA-CSIC), 33300 Oviedo, Spain; 5 Department of Pediatrics, School of Medicine, University of Valencia, Valencia, Spain; 6 Pediatric Gastroenterology and Nutrition Section, Hospital Clínico Universitario Valencia, INCLIVA, Valencia, Spain; 7 Department of Functional Biology, University of Oviedo, 33006 Oviedo, Spain

**Keywords:** Microbiota, Mother–infant, Causal mediation analysis, Dietary indices, Gut diversity

## Abstract

**Purpose:**

Maternal diet shapes maternal and infant microbiota, influencing early-life health. This study assessed associations between maternal dietary indices and infant gut microbiota, and the mediating role of maternal core taxa.

**Methods:**

In the MAMI cohort, pregnancy diet was evaluated using the Modified Mediterranean Dietary Score (MMDS), Dietary Quality Index (DQI), Healthy Eating Index (HEI), and Dietary Inflammatory Index (DII). Perinatal data, clinical records, and infant gut microbiota (1 month postpartum, 16S rRNA sequencing) were analyzed using clustering, regression, and causal mediation analysis (CMA).

**Results:**

Among 104 mother–infant pairs, DII was inversely correlated with MMDS, HEI, and DQI. Higher MMDS (β = 0.785, *p* = 0.037) and lower DII (β = –0.783, *p* = 0.037) were associated with increased infant *Veillonella* relative abundance; MMDS was also positively linked to Shannon and Simpson diversity. In the maternal microbiota, *Coprococcus* correlated positively with MMDS and negatively with DII. CMA identified maternal *Coprococcus* as the strongest mediator, linking MMDS to lower infant *Veillonella* (a × b = –0.15) and DII to higher infant *Veillonella* (a × b = 0.15).

**Conclusions:**

A Mediterranean-style pregnancy diet was associated with greater infant gut diversity and, via maternal *Coprococcus*, lower *Veillonella* abundance, whereas a pro-inflammatory diet showed the opposite pattern. These findings highlight both direct and microbiota-mediated pathways linking maternal nutrition to early microbial programming.

*Clinical trial registry number*: registration ID: NCT03552939 (date record study 2018–06-10). URL: https://clinicaltrials.gov/study/NCT03552939.

**Supplementary Information:**

The online version contains supplementary material available at 10.1007/s00394-026-03909-9.

## Background

Alteration on the infant gut microbiota have been linked to immune-related conditions, and higher risk of non-communicable diseases (NCDs) with implications for health programming [1–5]. Early life microbial colonisation and assembly is known to beshaped by several perinatal factors, including maternal nutritional status, mode of delivery, antibiotic use and breastfeeding practices [6, 7]. The maternal microbiota is the main microbial source for the infant gut, that is characterised by lower microbial diversity and higher prevalence of *Bifidobacterium* members [8–10], which have been also reported to be influenced by the maternal diet [11–15]. Where maternal gut *Bifidobacterium* and *Bacteroides* strains are vertically transferred and they are adapted to persist in the infant gut [16–18] and also, breastfeeding practices influence the microbiota [9, 19] and is linked to reduce risk of infectious diseases and allergies development later in life [20–24].

The maternal diet have been reported to modulate some bioactive compounds present in human milk as HMO [25], microRNA [26] and also, microbiota [27] with potential consequences on the infant microbiota evolution. Although the exact mechanisms is unclear, some studies have reported. For example, maternal intake of polyphenols can bind to and alter cell membrane functions, inhibiting bacterial growth [28], and the anaerobic fermentation of dietary fibers and HMO produces short-chain fatty acids and other metabolites that impact microbial composition and activity [29]. Consequently, maternal diet significantly shapes both maternal and infant microbiota, impacting infant health and supporting the 'thrifty phenotype hypothesis’ [30].

Until now, literature has often studied maternal dietary exposure to specific foods, such as fish oil [12], salmon [31], and nutrients including omega-3 fatty acids [32], vitamin D [13, 33], and fat intake [34], as well as food groups or clusters [11, 35, 36] in relation to infant gut microbiota. However, there is limited evidence [14] analysing the impact that dietary patterns may have on the microbiota in children.

**Fig. 1:**
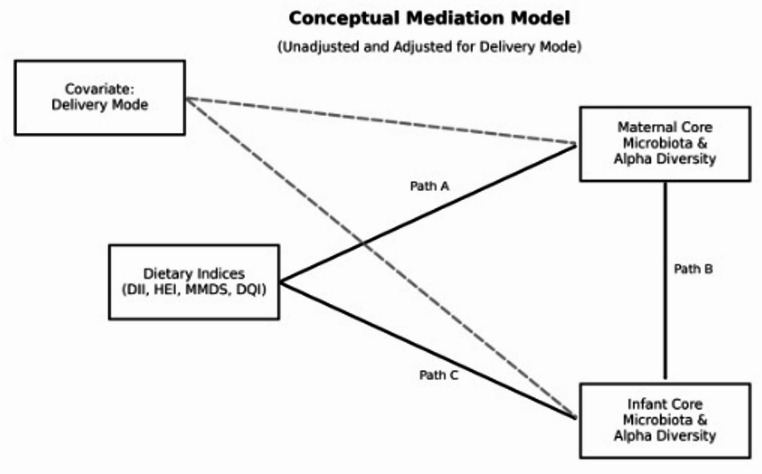
Conceptual Mediation Model representing the hypothesized paths between maternal dietary indices and infant gut outcomes. **Path A** illustrates the association between maternal Dietary Indices (DII, HEI, MMDS, DQI) and maternal core microbiota and alpha diversity (mediator). **Path B** represents the link from maternal core microbiota and alpha diversity to infant core microbiota and alpha diversity (outcome). **Path C** denotes the direct effect of maternal dietary indices on infant gut microbial outcomes. Dashed arrows indicate covariate adjustment for delivery mode. Maternal and infant core microbiota are defined as taxa present in at least 50% of samples above 0.001% relative abundance

In this scenario, we aim to assess the influence of maternal diet on infant core microbiota measured by several dietary indices and to investigate the potential mediation by maternal core gut microbiota of the effect of maternal diet on the core gut microbiota of exclusively breastfed infants (Fig. 1).

## Materials and methods

### Study design and volunteers

**Fig. 2:**
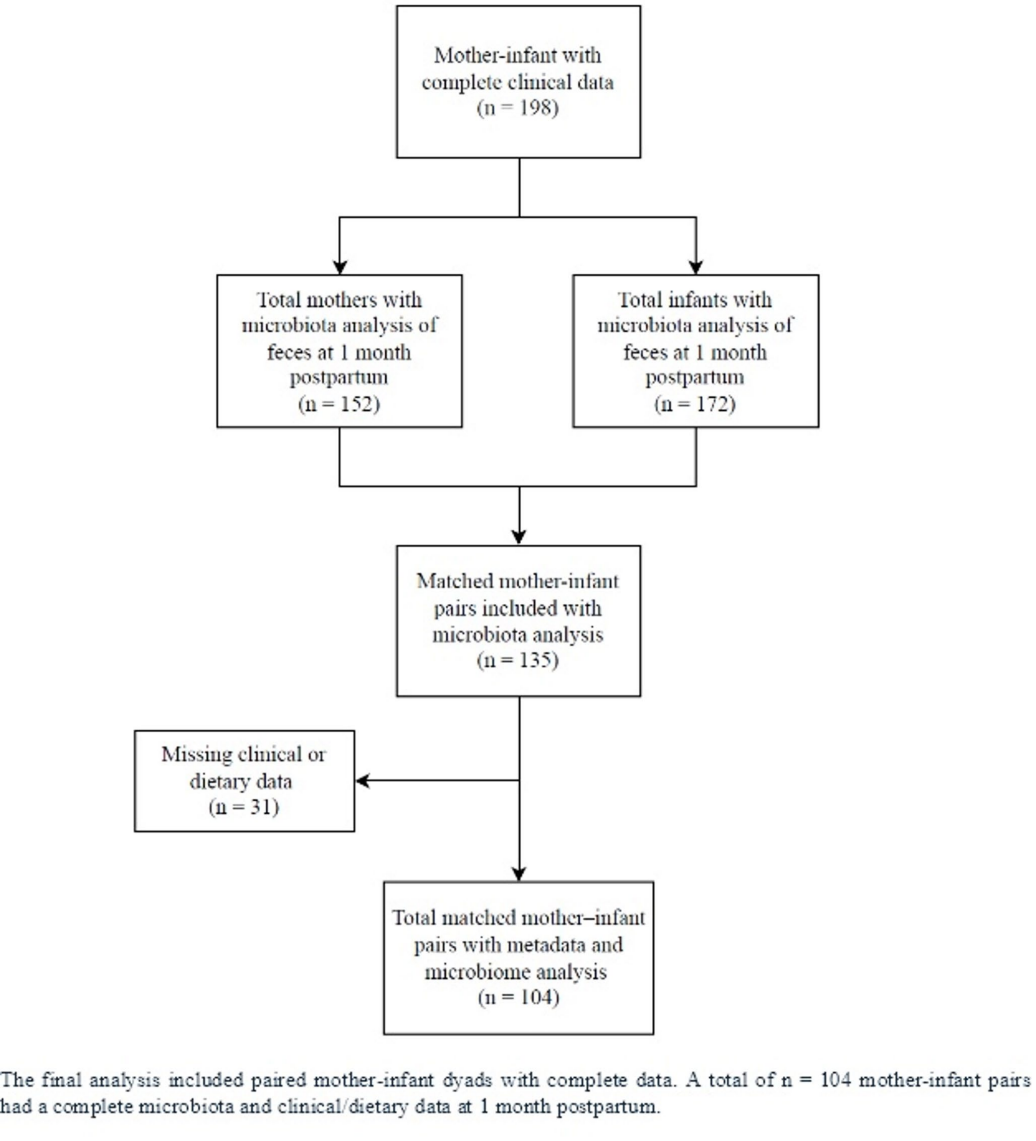
Participants flowchart. The final analysis included paired mother-infant dyads with complete data. A total of n = 104 mother-infant pairs had a complete microbiota and clinical/dietary data at 1 month postpartum

This study is a cross-sectional analysis involving mother–infant dyads from the Maternal Microbiome (MAMI) cohort, as outlined in the flowchart (Fig. 2). Maternal and infant samples were collected at 1 month postpartum (n = 104 pairs). The following maternal-infant clinical parameters were collected: gestational age, pregestational BMI, gestational weight gain, antibiotics during pregnancy, antibiotics during labor, delivery mode, sex, weight at birth, and antibiotics 7 to 15 days post-partum, amongst others. Maternal-infant biosamples, feces and human milk, were collected at 1 month post-partum, respectively. Details about the recruitment, sampling methods and storage were described previously [37]. In brief, the recruitment was conducted in primary health centers and hospitals. Eligible mothers were over 18, had a healthy pregnancy, and spoke Spanish, excluding those with certain medical conditions or on medication. Participants received oral and written information about the study, and written consent was obtained from each participant. The study was approved by the Ethical Committee of the Hospital Clínico Universitario Valencia, Spain and by CSIC Ethics Committee.

This study followed the STROBE-nut (Strengthening the Reporting of Observational Studies in Epidemiology—Nutrition) [38] reporting guidelines for observational studies (Electronic Supplementary Material Table S1).

### Maternal dietary assessment and diet-related indexes

Dietary records were collected during the first weeks after birth by a nutritionist using a 140-item food frequency questionnaire (FFQ) as described by [39]. FFQ information was analyzed for the energy and daily intake of macro- and micronutrients by using the nutrient Food Composition Tables developed by the Centro de Enseñanza Superior de Nutrición Humana y Dietética (CESNID) [40]. Based on a previously validated FFQ [14], specific dietary indices were calculated, including the Dietary Inflammatory Index (DII), associated with adverse health outcomes, and others linked to positive health effects, such as the Diet Quality Index (DQI), the Modified Mediterranean Diet Score (MMDS), and the Healthy Eating Index (HEI):

#### The modified dietary inflammatory index (DII)

The DII quantifies the inflammatory potential of diets using a comprehensive literature review [41]. This method involved calculating 35 out of 45 parameter-specific inflammatory effect score. Using IBM SPSS Statistics 25.0 [42], individual dietary intake data were converted to Z-scores, percentile scores, and finally centered percentile scores, which were multiplied by food parameter effect scores. Summing these scores provided the overall DII, ranging from − 8.87 (anti-inflammatory) to 7.98 (pro-inflammatory).

**Table 1:** Participant maternal and infant clinical characteristics

Characteristics	1 month postpartum (n = 104)
*Maternal characteristics*	
Gestational age (weeks)*	40 [39, 40]
Pregestational BMI, kg/m2*	22.46 [20.5–24.05]
Gestational weight gain, kg*	12 [10–15]
*Antibiotics during pregnancy (n)*	
Yes	29 (29/104, 27.88%)
No	75 (75/104, 72.12%)
*Antibiotics during labor (n)*	
Yes	29 (29/104, 27.88%)
No	75 (75/104, 72.12%)
*Delivery mode (n)*	
C-section	27 (27/104, 25.96%)
Vaginal	77 (77/104, 74.04%)
Infant characteristics †	
*Sex (n)*	
Female	60 (60/104, 57.69%)
Male	44 (44/104, 42.31%)
Weight at birth (kg)	3.29 [3–3.58]
*Antibiotics 7 to 15 days post-partum*	
Yes	6 (6/104, 5.77%)
No	98 (98/104, 94.23%)
*Maternal dietary characteristics*	
DII*	0.6 [− 2.25–2.52]
DQI*	64 [55, 58–68]
MMDS*	4 [3–5]
HEI*	71.88 [65.39–76.27]

*Median [Q1-Q3]

^†^All infants were exclusively breastfed from birth

#### The diet quality index (DQI)

The DQI score, ranging from 0 to 100, was used to evaluate diet quality by matching individual dietary intake data with recommended servings and nutrient intakes, then categorizing them into food groups. Each food group's intake was scored on a scale, with higher scores indicating better dietary quality, using the scoring criteria from Table 2 of Mariscal-Arcas et al. [43]. Components such as variety, adequacy, moderation, and overall balance were assessed. Scores for each category were calculated based on specific criteria and summed to provide an overall DQI score. To integrate the DQI calculation in IBM SPSS Statistics 25.0, each component in the sample was evaluated by assigning a value of 0 to 3, 0 to 5, or 0 to 6, as appropriate, then scores were added to obtain the total DQI score for each individual.

**Table 2:** Linear regression results showing statistically significant associations (*p* < 0.05) between dietary indices and bacterial genera

Bacteria and diversity	Diet	β [95% CI]	*P* value
*Members of infant gut microbiota core (genus level)*			
*Veillonella*	DII	-0.783 [-1.520, -0.047]	0.037
*Veillonella*	MMDS	0.785 [0.049, 1.522]	0.036
*Infant diversity indices*			
Shannon	MMDS	0.211 [0.018, 0.403]	0.032
Simpson	MMDS	0.194 [0.001, 0.387]	0.048
*Members of maternal gut microbiota core (genus level)*			
*Romboutsia*	DII	-0.439 [-0.869, -0.009]	0.045
*Coprococcus*	DII	-0.655 [-1.155, -0.155]	0.011
*Butyricicoccus*	DQI	0.715 [0.096, 1.334]	0.023
*Faecalibacterium*	DQI	0.432 [0.037, 0.827]	0.032
*Fusicatenibacter*	HEI	0.647 [0.072, 1.223]	0.027
*Incertae_Sedis*	HEI	-0.395 [-0.715, -0.076]	0.015
*Faecalibacterium*	HEI	0.442 [0.049, 0.835]	0.027
*Bifidobacterium*	HEI	-0.577 [-1.082, -0.073]	0.025
*Eubacterium coprostanoligenes* group	MMDS	-0.416 [-0.8, -0.031]	0.034
*Anaerostipes*	MMDS	0.381 [0.069, 0.692]	0.017
*Monoglobus*	MMDS	0.482 [0.108, 0.856]	0.011
*Coprococcus*	MMDS	0.658 [0.158, 1.158]	0.01
*Bifidobacterium*	MMDS	-0.549 [-1.053, -0.045]	0.033
*Christensenellaceae R.7* group	MMDS	-0.662 [-1.072, -0.251]	0.001
*Maternal diversity indices*			
Chao1	HEI	0.204 [0.011, 0.397]	0.038
Shannon	HEI	0.249 [0.058, 0.441]	0.011
InvSimpson	HEI	0.269 [0.0788, 0.459]	0.006
Shannon	MMDS	0.219 [0.028, 0.412]	0.025
InvSimpson	MMDS	0.242 [0.051, 0.432]	0.013
Simpson	MMDS	0.205 [0.012, 0.398]	0.037

Each row displays the estimated regression coefficient and raw*p*-value for the relationship between one of four dietary scores.DII, MMDS, DQI, and HEI and the relative abundance of a microbialtaxon.Positive coefficients indicate a direct association, while negative valuesreflect inverse relationships. FDR-adjusted *p*-values were no longer significant after correction areindicated accordingly

#### The modified mediterranean diet score (MMDS)

The MMDS ranges from 0 to 10 and evaluates adherence to a Mediterranean diet [44]. For each component typically consumed in high quantities (vegetables, legumes, fruit/nuts, fish/seafood, and cereals). To integrate the MMDS calculation in IBM SPSS Statistics 25.0, the population medians were obtained for each variable to be analyzed (except alcohol). Most variables can be taken directly from the database, while others had to be created (e.g., MMDS_fruit_nuts, MMDS_fish_seafood, and MMDS_fat_ratio, calculated as the the fraction of monounsaturated and polyunsaturated fatty acids relative to saturated fatty acids). The values below the median were recoded as 0 and values above the median as 1. For meat, meat products and dairy products, the coding was reversed. For alcohol, the scores were based on whether individuals fall within the recommended range (1 if within the range, 0 if outside). Finally, we added the scores of all the variables to obtain the total MMDS score for each mother.

#### The healthy eating index (HEI)

The HEI-2015 evaluates diet quality based on 13 components, scored from 0 to 100, where a higher score indicates a healthier diet, and a lower score suggests a less healthy diet [45]. The calculation relied on densities, the amount of a dietary component per 1,000 kcal. These densities were compared to established standards. Regarding the calculation, to integrate the HEI-2015 calculation in IBM SPSS statistics 25.0, the servings for scoring each individual from the provided Table 1 from Krebs-Smith et al. [45] were obtained. Next, the food group division as stablished in Electronic Supplementary Material Table S2. was used to categorize foods groups. For each food group, the amount of cups, ounces, grams, or percentage consumed by the individual was determined. These scores are detailed in Krebs-Smith et al. [45]. Finally, the total scores were added to determine each individual's overall HEI score.

### Biomaterials and microbiota profiling by 16S amplicon sequencing

Fecal samples from mothers were collected at 1 month postpartum. Infant fecal samples were collected at the 1-month follow-up visit. These samples were gathered one month after childbirth using a standardized procedure well-documented in previous studies [46]. In summary, feces from both mothers and infants were collected in sterile containers, infant fecal samples were collected at home by their parents who were previously trained by clinical personnel in the health care centres where they were enrolled, morning collection was recommended. Briefly, fecal samples were deposited in provided sterile containers and immediately kept at − 20 °C, before the final storage at − 80 °C until further analysis.

Fecal DNA was extracted as previously described [15] using the Master-Pure DNA Extraction Kit (Epicentre, WI, USA) with physical and enzymatic modifications. Microbial profiling targeted the V3–V4 region of the 16S rRNA gene and was sequenced on an Illumina MiSeq platform (2 × 300 bp, MiSeq Reagent Kit v3) at FISABIO (Valencia, Spain). Sequence processing was done with DADA2 [47], and taxonomic assignment used the Silva v132 database [48]. Genus-level relative abundances and alpha-diversity metrics (Observed, Chao1, Shannon, Simpson, InvSimpson, Fisher) were calculated using the Phyloseq package (v4.3.1) [49]. Tables at genus taxonomical level and alpha-diversity indexes were used in this study to combine with the dietary indexes. Prior to regression analysis, maternal and infant bacterial counts were transformed into relative abundances. Subsequently, the core microbiota for both maternal and infant groups was defined as taxa present in at least 50% of the samples above 0.001% relative abundance. Then centered log-ratio transformed (CLR) was applied to bacterial relative abundances.

### Data processing and statistical methods

Complete data, including clinical, dietary, and gut microbiota information, was available for 104, mother-infant pairs at 1 month postpartum, respectively, as described in Fig. 2. Data preprocessing involved z-score normalization of dietary indices, the individual food components of those indices, and diversity indices, as well as centered log ratio (CLR) transformation of bacterial relative abundances.

A Principal Component Analysis (PCA) was performed to explore the relationships among various maternal dietary indices, including the DQI, DII, MMDS, and HEI. This analysis was executed using the prcomp function from the stats package in R [50], which facilitated the reduction of dimensionality by transforming the original variables into a new set of uncorrelated principal components. The PCA results were visualized through a biplot, which was generated using the ggplot2 [51] and ggpubr [52] libraries. Additionally, cluster analysis was incorporated by applying the k-means method to the PCA scores to discern distinct nutritional behavior patterns. The optimal number of clusters was determined using the elbow method, implemented via the fviz_nbclust function from the factoextra package [53].

### Linear regression models

To explore associations between dietary quality and gut microbiota composition, we performed a series of linear regression models using each dietary score, DII, MMDS, DQI, and HEI, as independent variables. Microbial taxa (normalized relative abundances via CLR transformation) were treated as dependent variables in univariate models. We used the statsmodels Python package [54] to fit ordinary least squares (OLS) regression models of the form genus (core member of the maternal and infant gut microbiota) ~ diet score, for each combination of microbial taxon and dietary index. Raw *p*-values were calculated for each regression, and false discovery rate (FDR) correction was applied across all tests using the Benjamini–Hochberg method to account for multiple comparisons. Associations with *p* < 0.05 before and after correction were considered for further interpretation. All analyses were conducted in Python (v3.12.7) [55] using pandas [56], statsmodels [54], statsmodels.stats.multitest [54], and joblib [57] for parallel computation.

### Causal mediation analysis (CMA)

To investigate whether the elements previously identified as significant in preliminary univariate regressions (*p* < 0.05) were associated with infant gut core microbiota and diversity, we conducted a CMA using a two-model regression framework (Electronic Supplementary Material Table S3).

For each selected maternal taxon as a potential mediator, we fit two regression models:

1. Mediator model:

$$Mi= \alpha 1+ \beta 1Xi+ \beta 2Ci+ \varepsilon 1$$

where MiM_iMi is the CLR-transformed abundance of the mediator taxon, XiX_iXi is the standardized maternal dietary score (e.g., MMDS), and CiC_iCi is the covariate for delivery mode.

- a-path (a = β1) represents the effect of the dietary score on the mediator taxon.

2. Outcome model:

$$Yi= \alpha 2+ \gamma 1Xi+ \beta 3Mi+ \beta 4Ci+ \varepsilon 2$$

where YiY_iYi is the CLR-transformed outcome (e.g., infant Simpson diversity or *Veillonella*), MiM_iMi is the mediator taxon, and CiC_iCi is the delivery mode covariate.

- b-path (b = β3) represents the effect of the mediator on the outcome, controlling for the dietary score.
- Direct effect (c′-path) (γ1) captures the effect of the dietary score on the outcome independent of the mediator.

The indirect effect was calculated as the product of the a and b paths (a × b), quantifying the mediation effect of each taxon. The total effect was derived as the sum of the direct and indirect effects:

$$Total Effect= \gamma 1+(a\times b)$$

To assess the significance of the indirect effect, we employed nonparametric bootstrapping with 1,000 iterations to generate empirical 95% confidence intervals and two-tailed pseudo p-values. Only models with complete data for all included variables were used. All analyses were adjusted for delivery mode.

## Results

### Maternal and infant characteristics and gut microbiota core one month post-partum

General characteristics of participants are available in Table 1. This study evaluated maternal and infant characteristics considering one-month maternal and infant gut microbiota core. Approximately 25% of the deliveries were via cesarean section, and the majority were vaginal (74%). Infant gender distribution was more female than male. Antibiotic use during pregnancy and labor was reported in about 28 of the cases. Maternal dietary indices such as DII, DQI, MMDS, and HEI showed minimal variation over the time points.

### Maternal dietary indices explain different patterns.

**Fig. 3:**
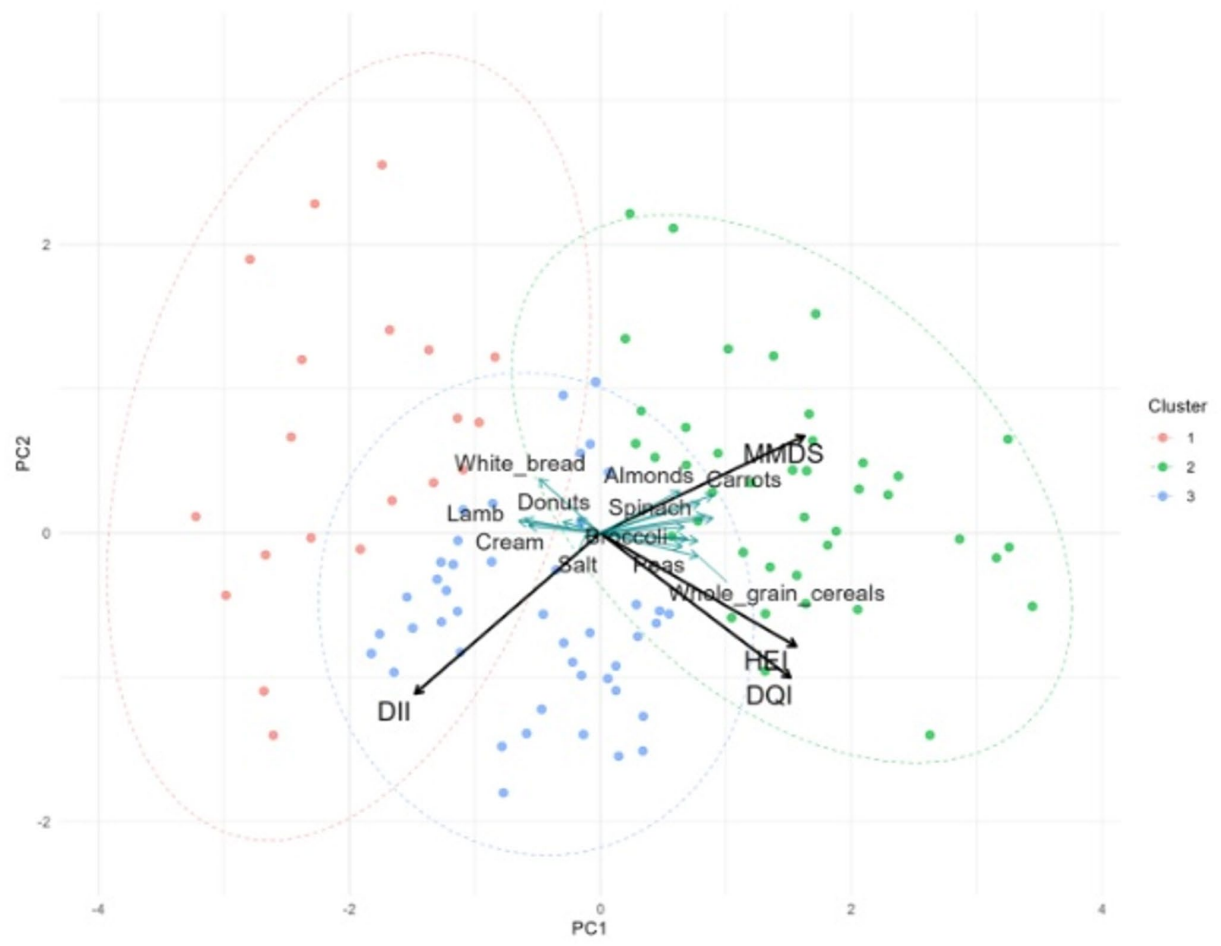
PCA biplot illustrating sample clustering and the contributions of dietary indices (black arrows) and the union of food items ranked among the eight most positively correlated variables for each dietary index (turquoise arrows). Samples are colored according to dietary pattern clusters (K-means, K = 3), with 95% confidence ellipses illustrating the separation among clusters. Arrow directions indicate the alignment of variables with the principal components

K-means clustering revealed three distinct dietary patterns, characterized by higher scores on the MMDS, elevated DQI and HEI, and a predominance of DII. The PCA biplot (Fig. 3) illustrates the relationships among dietary indices, with DQI and HEI closely aligned, reflecting their positive correlation (r = 0.59), and shared focus on overall dietary quality. In contrast, DII is negatively correlated with MMDS (r = –0.61), HEI (r = –0.40), and DQI (r = –0.29). These patterns are further supported by food-level correlations, where pro-inflammatory foods (e.g., processed meats, refined products) align with DII, and anti-inflammatory, nutrient-dense foods (e.g., legumes, fruits, vegetables, whole grains) align with MMDS, DQI, and HEI.

### Maternal diet and infant gut microbiota linear regressions

Significant associations were observed between maternal and infant gut microbiota composition and several dietary indices (Table 2). In infants, *Veillonella* was inversely associated with the DII (β =  − 0.783, 95% CI: [− 1.520, − 0.047], *p* = 0.037) and positively associated with the MMDS (β = 0.785, 95% CI: [0.049, 1.522], *p* = 0.037). Infant alpha diversity metrics also showed positive associations with MMDS, including Shannon diversity (β = 0.211, 95% CI: [0.018, 0.403], *p* = 0.032) and Simpson diversity (β = 0.194, 95% CI: [0.001, 0.387], *p* = 0.049).

Among maternal gut microbiota genera, *Coprococcus* was inversely associated with DII (β =  − 0.655, 95% CI: [− 1.155, − 0.155], *p* = 0.011), and positively with MMDS (β = 0.658, 95% CI: [0.159, 1.159], *p* = 0.010). Additional taxa, including *Fusicatenibacter*, *Faecalibacterium*, *Incertae Sedis*, and *Bifidobacterium*, showed significant relationships with HEI and other diet scores. Maternal diversity indices such as Chao1 (β = 0.204, 95% CI: [0.011, 0.397], *p* = 0.038), Shannon (β = 0.249, 95% CI: [0.059, 0.441], *p* = 0.011), and Inverse Simpson (β = 0.269, 95% CI: [0.079, 0.459], *p* = 0.006) were also positively associated with HEI.

However, none of the associations remained statistically significant after FDR correction, indicating potential Type I error due to multiple testing.

### CMA of maternal diet and infant gut microbiota: the mediating role of maternal microbiota

To evaluate whether specific maternal microbial taxa mediated the relationship between DII and the relative abundance of *Veillonella*, we conducted CMA across core maternal genera and diversity indices. Among the top eight coefficients ranked by the magnitude of the indirect effect (a × b), three genera; *Coprococcus*, *Lachnospiraceae FCS020* group, and *Eubacterium coprostanoligenes group*, showed consistent and relatively large mediation effects (unadjusted a × b ≥ 0.12), with comparable results after adjusting for delivery mode.

**Fig. 4:**
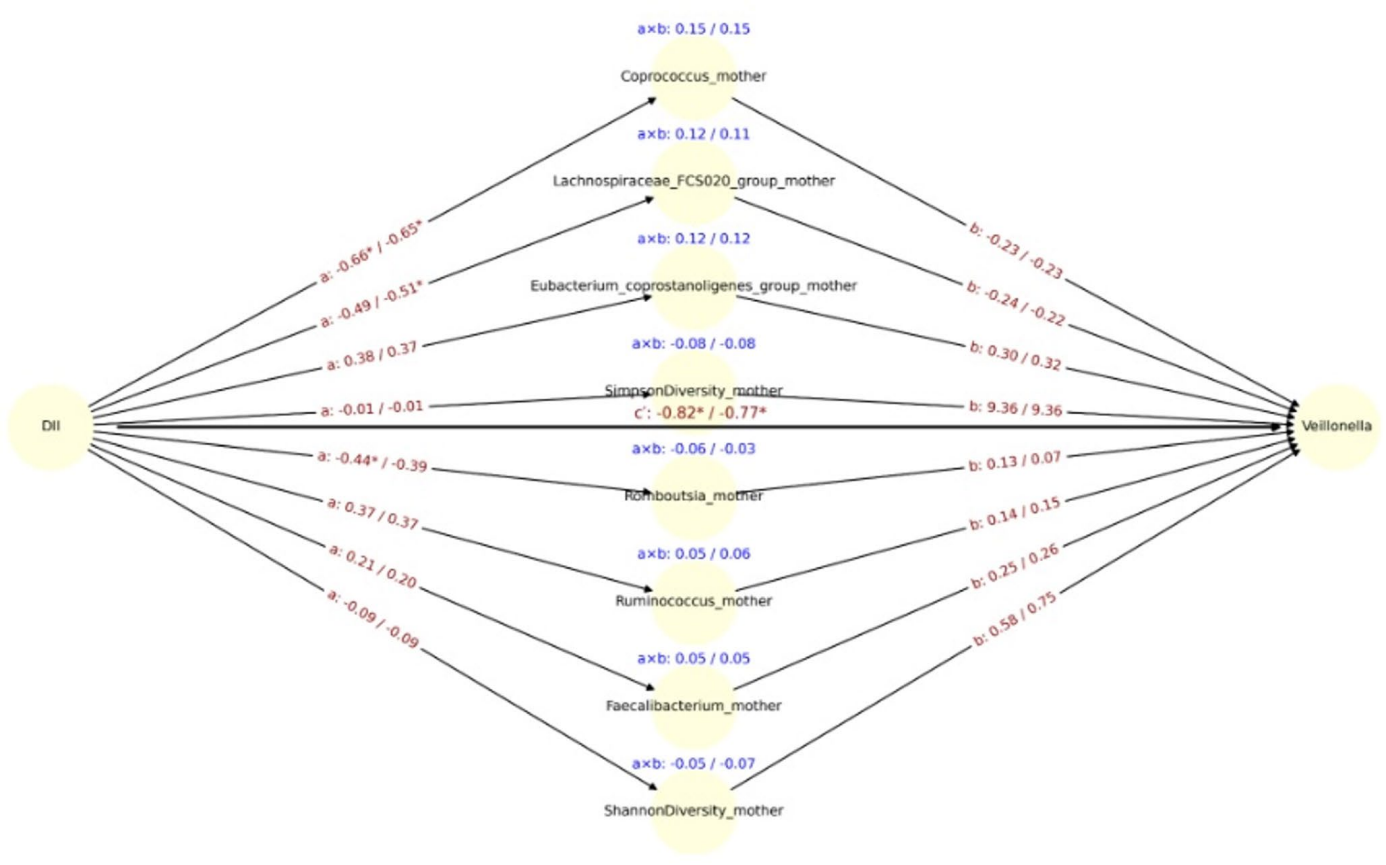
Mediation path diagram illustrating associations between maternal DII and the relative abundance of *Veillonella* in infants, via maternal gut microbial genera. Each node represents a mediator genus (center), the exposure (DII, left), or the outcome (*Veillonella*, right). Arrows represent estimated regression coefficients from the mediation models: a-path: effect of DII on the mediator genus, b-path (right): effect of the mediator genus on *Veillonella*, c′-path (center): direct effect of DII on *Veillonella*, adjusted for the mediators. For each path a, b, and a × b (indirect effect), both unadjusted and delivery-mode, adjusted estimates are shown as: estimate_unadj / estimate_adj. Asterisks (*) indicate statistical significance (*p* < 0.05) for the corresponding coefficient. The c′path at the center reflects the mean direct effect of DII on *Veillonella* across all models. Indirect effects (a × b) are displayed in blue above each mediator. Only the top 8 genera with the largest unadjusted indirect effects (by absolute value) are shown

Notably, *Coprococcus* exhibited the strongest indirect effect (a × b = 0.15), driven by a strong inverse association between DII and *Coprococcus* (a = –0.66, *p* < 0.05), and a negative link between *Coprococcus* and *Veillonella* (b = –0.23). Adjustment for delivery mode did not substantially alter these coefficients (a × b = 0.15; a = –0.65, b = –0.23) (Fig. 4).

**Fig. 5:**
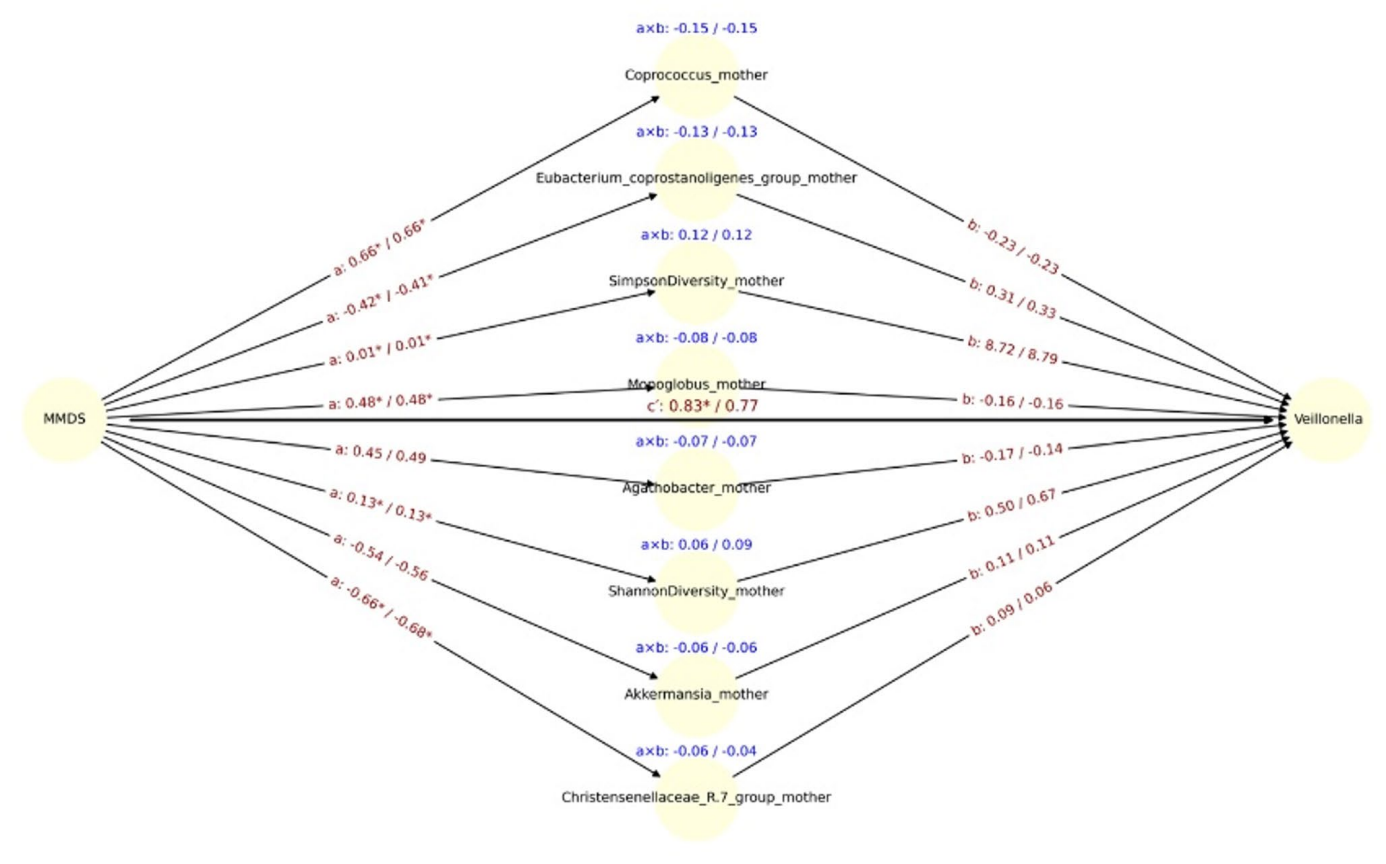
Mediation path diagram illustrating associations between maternal MMDS and the relative abundance of *Veillonella* in infants, via maternal gut microbial genera. Each node represents a mediator genus (center), the exposure (MMDS, left), or the outcome (*Veillonella*, right). Arrows represent estimated regression coefficients from the mediation models: a-path: effect of MMDS on the mediator genus, b-path (right): effect of the mediator genus on *Veillonella*, c′-path (center): direct effect of MMDS on *Veillonella*, adjusted for the mediators. For each path a, b, and a × b (indirect effect), both unadjusted and delivery-mode, adjusted estimates are shown as: estimate_unadj / estimate_adj. Asterisks (*) indicate statistical significance (p < 0.05) for the corresponding coefficient. The c′path at the center reflects the mean direct effect of DII on *Veillonella* across all models. Indirect effects (a × b) are displayed in blue above each mediator. Only the top 8 genera with the largest unadjusted indirect effects (by absolute value) are shown

**Fig. 6:**
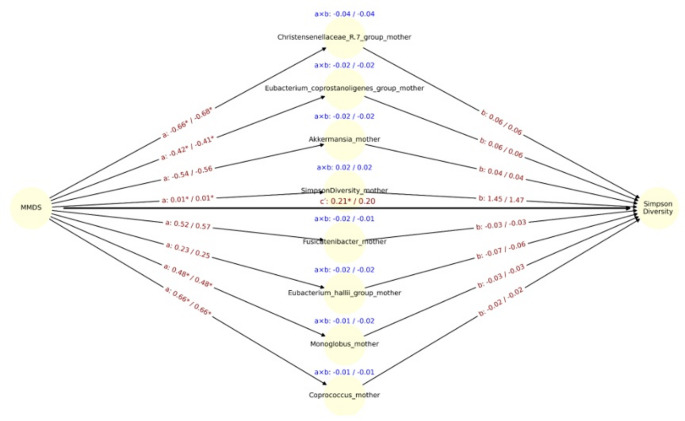
Mediation path diagram illustrating associations between maternal MMDS and the relative abundance of Simpson diversity in infants, via maternal gut microbial genera. Each node represents a mediator genus (center), the exposure (MMDS, left), or the outcome (Simpson diversity, right). Arrows represent estimated regression coefficients from the mediation models: a-path: effect of MMDS on the mediator genus, b-path (right): effect of the mediator genus on Simpson diversity, c′-path (center): direct effect of DII on Simpson diversity, adjusted for the mediators. For each path a, b, and a × b (indirect effect), both unadjusted and delivery-mode, adjusted estimates are shown as: estimate_unadj / estimate_adj. Asterisks (*) indicate statistical significance (*p* < 0.05) for the corresponding coefficient. The c′path at the center reflects the mean direct effect of DII on Simpson diversity across all models. Indirect effects (a × b) are displayed in blue above each mediator. Only the top 8 genera with the largest unadjusted indirect effects (by absolute value) are shown

**Fig. 7:**
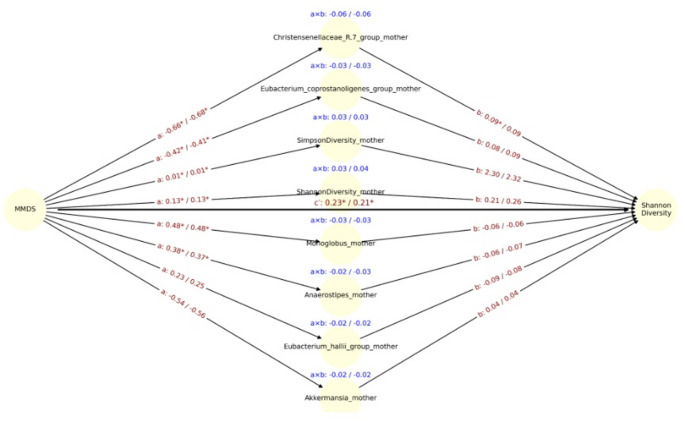
Mediation path diagram illustrating associations between maternal MMDS and the relative abundance of Shannon diversity in infants, via maternal gut microbial genera. Each node represents a mediator genus (center), the exposure (MMDS, left), or the outcome (Shannon diversity, right). Arrows represent estimated regression coefficients from the mediation models: a-path: effect of MMDS on the mediator genus, b-path (right): effect of the mediator genus on Shannon diversity, c′-path (center): direct effect of DII on Shannon diversity, adjusted for the mediators. For each path a, b, and a × b (indirect effect), both unadjusted and delivery-mode, adjusted estimates are shown as: estimate_unadj / estimate_adj. Asterisks (*) indicate statistical significance (*p* < 0.05) for the corresponding coefficient. The c′path at the center reflects the mean direct effect of DII on Shannon diversity across all models. Indirect effects (a × b) are displayed in blue above each mediator. Only the top 8 genera with the largest unadjusted indirect effects (by absolute value) are shown

The MMDS was significantly associated with lower maternal *Christensenellaceae* abundance (path a: β =  −0.66, *p* = 0.0014), and *Christensenellaceae* was positively associated with infant Shannon diversity (path b: β = 0.09, *p* = 0.047), although after adjustment for delivery mode significance was lost (Fig. 5). In contrast, although diet was significantly associated with maternal microbiota, no significant mediators were identified for Simpson diversity or *Veillonella* abundance, as no microbial genera demonstrated simultaneous significance on both path a and path b (Fig. 6). The direct effect of the DII on *Veillonella* abundance remained statistically significant after adjusting for delivery mode (cʹ =  − 0.82 unadjusted / − 0.77 adjusted, *p* < 0.05 for both models). Similarly, in models using the MMDS as the exposure, direct effects were also significant for *Veillonella* (c′ = 0.83 / 0.77, *p* < 0.05 for unadjusted model), Simpson diversity (c′ = 0.21 / 0.20, p < 0.05 for unadjusted model), and Shannon diversity (c′ = 0.23 / 0.21, *p* < 0.05 for both models) (Fig. 7). These findings suggest that the observed associations between maternal diet and infant microbiota or diversity metrics are not fully mediated by maternal gut microbial genera and may reflect direct influences of maternal diet or dietary-associated host factors.

## Discussion

We observed that maternal diet influences the infant gut microbiota at one month of age, and this effect is partially mediated by maternal microbiota changes related to diet during gestation. Specifically, the *Veillonella* genus, Shannon and Simpson diversity indices in the infant gut were partially mediated by members of the maternal gut core and diversity. A pro-inflammatory maternal diet was associated with a direct reduction in *Veillonella* abundance. At the same time, maternal gut microbiota at the genus level partially mediated this effect. For instance, genera such as *Coprococcus* appeared to partially mediated an increase in *Veillonella* in response to DII, while others, such as *Romboutsia*, partially mediated a decrease. Additionally, opposing effects of maternal MMDS and DII on *Veillonella* were observed in the CMA model. MMDS was positively associated with Shannon and Simpson diversity indices. However, when mediation models included specific members of the maternal gut microbiota, opposing effects emerged, further highlighting the complexity of microbial interactions.

This is the second study to use dietary indices in the MAMI cohort [14]. Compared to other populations, our cohort shows similar adherence to dietary indices (Table 3). In terms of the HEI, other populations have also shown high adherence, indicating a lower preference for Western dietary patterns [58–61]. However, one study noted that 80% of its U.S. cohort exhibited lower adherence to the HEI [62]. Regarding the DII, which measures the inflammatory potential of dietary components, the scores are distributed more evenly across studies, reflecting varied dietary influences within the same populations [63].

**Table 3:** Table comparison of studies reporting maternal dietary index during gestation

References	Population	Sample size (n)	Cohort	DII*	DQI*	MMDS*	HEI*
Rodriguez et al. [66]	Spain	54	INMA	NA	NA	NA	48–51
Shapiro et al. [67]	United States	1079	The Healthy Start	NA	NA	NA	54.2 (13.6)
Asadi et al. [68]	Iran	350	NA	NA	NA	NA	70.1 (11.11)
Sivula et al. [69]	Finland	1330	KuBiCo	− 0.095 (2.3)	NA	NA	58.1 (10.1)
Monthé et al. [70]	United States	1459	Project Viva	− 2.6 (1.4)	NA	4.6 (2)	61 (10)
Almulla et al. [71]	United Arab Emirates	122	Mutaba’ah Study	NA	NA	4.5 (0.5)	62.5 (2.5)
Garcia et al. [72]	Spain	86	MAMI	NA	NA	8.8 (1.7)	NA
Leda et al. [73]	Spain	2461	INMA	NA	NA	3.8 (1.6)	NA
Leda et al. [73]	Greece	889	RHEA	NA	NA	4 (1.7)	NA
Hayat et al. [74]	Iran	90	NA	NA	52.64(7.94)	NA	NA
Souza et al. [75]	Brazil	260	NA	0.04 (3.2)	68.82 (80)	NA	NA
This study	Spain	246	MAMI	0.58 (4.61)	64.5 (11.41)	4 (1)	71.92 (11)

*NA* not available, *DII* dietary inflammatory index, *MMDS* modified mediterranean dietary score, *HEI* healthy eating index, *MAMI* maternal microbe cohort, *INMA* Infancia y Medio Ambiente—environment and childhood, *RHEA* mother–child cohort in Crete, and *KuBiCo* Kuopio birth cohort

*Values correspond to median (interquartile range)

The lactate-utilizing bacterium *Veillonella* is a prominent early colonizer of the infant gut microbiota. Studies have identified vertical transmission events of *Veillonella* strains, particularly via human milk (72) and maternal fecal samples, contributing to initial gut colonization [64]. However, in our cohort, Spearman correlation tests between maternal and infant *Veillonella* gut abundance at one month postpartum did not reveal a significant association (ρ (rho) = –0.016, *p* = 0.872). Moreover, *Veillonella* has not been detected as the most frequently transmitted bacterial genera [65].

Despite this, *Veillonella* plays a beneficial role in shaping the infant gut microbial community. Its metabolic functionality, primarily driven by the methylmalonyl-CoA pathway along with other reductive pathways, enables energy production under the low-redox conditions typical of the infant colon. This allows *Veillonella* to metabolize lactate, derived from other microbial taxa and dietary sources, into short-chain fatty acids (SCFAs) such as propionate and acetate, which support gut health [76]. A decreased abundance of *Veillonella* has been associated with a higher risk of asthma [77]. As an obligate anaerobic, Gram-negative bacterium, *Veillonella* contributes to gut homeostasis, playing a key role in immune modulation, epithelial barrier integrity, and luminal pH regulation [78, 79].

Our mediation models show that maternal dietary patterns during pregnancy exert both direct and indirect influences on *Veillonella* abundance in infants at one month. The DII negatively impacted *Veillonella* levels, while a MMDS showed generally positive associations. These effects were partially mediated through specific members of the maternal gut microbiota at one month postpartum, including genera such as *Coprococcus*, *Romboutsia*, *Lachnospiraceae*, and *Eubacterium*, many of which are involved in upstream fermentation of complex carbohydrates [80–83] and can alter the availability of *Veillonella’s* primary substrate, lactate. These microbial shifts may also play a role in modulating infant gut microbial diversity, particularly in response to Mediterranean dietary patterns during pregnancy.

This reflects broader ecological and metabolic interdependencies: *Veillonella* thrives in environments where lactate is abundant and is likely to be in a metabolically active, quasi-exponential (log) phase under such conditions [84]. When lactate becomes limited, whether due to substrate scarcity or competitive uptake by co-colonizing taxa, *Veillonella* may shift to a stationary-like metabolic state, characterized by reduced SCFA production, stress-response gene expression, and metabolic reprogramming. Such phase-dependent shifts have been described in vitro, with *V. dispar* showing reduced propionate output and transcriptional changes in central metabolic genes under nutrient-limited conditions [78, 84].

In vivo, these dynamics are further shaped by strain-level variability. Different *Veillonella* strains differ in lactate transporter efficiency, SCFA production ratios, and biofilm formation capacity, all of which may influence their ability to colonize and persist in the infant gut [79, 85, 86]. Quorum sensing systems [87], although not well-characterized in *Veillonella*, may play a role in coordinating population-level behaviors such as biofilm formation and substrate utilization in response to local microbial density or host signaling molecules, and autoinducers, and may be affected indirectly by maternal microbial ecology.

Importantly, the maternal gut microbiota at one month postpartum may reflect longer-term dietary influences, which in turn modulate microbial metabolite profiles (e.g., lactate availability) that affect vertically transmitted or environmentally acquired *Veillonella* strains in the infant. The observed mediation by maternal genera and diversity indices reinforces the importance of microbial cross-feeding, ecological succession, and nutrient shaping in early life microbiota assembly, particularly for key fermenters like *Veillonella* that occupy metabolic niches downstream of primary saccharolytic fermenters, such as *Coprococcus*, *Eubacterium coprostanoligenes group*, *Agathobacter*, and *Christensenellaceae R-7 group* [80, 88, 89]*.*

Gestational adherence to a MMDS was positively associated with infant gut microbial diversity at one month, with effects partially mediated by the maternal core gut microbiota at one month postpartum. Key mediators included *Coprococcus*, *Eubacterium hallii group*, and *Christensenellaceae R-7 group*, fiber-fermenting taxa enriched by plant-based diets and known for SCFA production [90–92]. Although direct effects of MMDS on infant diversity were stronger, the microbial mediation paths suggest that postpartum maternal microbiota composition, shaped by earlier dietary exposures, contributes to infant ecological outcomes. Maternal adherence to a Mediterranean diet may therefore influence the early phases of microbial assembly. Although diversity is physiologically lower in early infancy than later in life, several cohorts suggest that relatively reduced diversity or delayed diversification during the first year is associated with adverse immune and allergic outcomes [93–95], rather than being beneficial per se. In this context, the MMDS associated increase in Simpson diversity at 1 month is likely to reflect a more advanced yet still age-appropriate maturation trajectory rather than dysbiosis, consistent with patterns typically observed in vaginally delivered versus caesarean-born infants, and in breastfed infants compared with those who discontinue breastfeeding [96, 97]. The accompanying enrichment of *Veillonella* spp., a characteristic early-life taxon that participates in cross-feeding networks, may indicate the emergence of a more functionally interconnected ecosystem shaped in part by maternal diet. However, as we did not measure microbiota function or long-term clinical outcomes, these implications remain speculative, and our findings should be interpreted as hypothesis-generating.

## Strengths and limitations

Several limitations of this study must be acknowledged. First, a comprehensive understanding of microbiota modulation and the identification of microbial signatures would ideally require a multi-omics approach [98–100]. Second, more data are needed to elucidate the initial seeding of the infant gut microbiota, particularly the role of other influencing factors such as human milk composition. Finally, the cross-sectional design limits causal inference, as it captures only a single time point, one month postpartum, providing a snapshot rather than a dynamic view of microbiota development [101].

Nevertheless, an strength of the study is its focus on gestational exposures, a time window considered etiologically relevant for early-life development [102–105]. Methodologically, the use of the 'abundance-occurrence' method to define the core microbiota aligns with accepted practices [106]. However, this approach tends to emphasize the most consistently detectable and abundant taxa, potentially overlooking less abundant but potentially biologically significant taxa.

The application of CMA is another strength, as it enables exploration of potential biological mechanisms, particularly highlighting the maternal microbiota as a mediator of the relationship between maternal diet and infant gut microbiota [107–109].

Furthermore, the study utilized multiple dietary indices to assess maternal dietary intake, which enhances the robustness of dietary characterization and helps to address some methodological biases [110, 111]. However, dietary data were obtained via a FFQ administered during the early postpartum period, relying on retrospective recall of the previous year’s diet. This introduces recall bias and measurement noise, limiting the precision of dietary exposure assessment [112].

The dietary indices were based on international standards, which, while comprehensive, may not fully capture local dietary patterns. Given that the study population was based in a Mediterranean region (Valencia, Spain), this raises concerns about external validity and the generalizability of the findings to other populations or food environments.

Another limitation lies in the 16S rRNA gene sequencing approach used for microbial profiling. While it allows taxonomic classification, it has known limitations, including potential false positives, with up to 20% of predicted genera possibly being incorrect when using the SILVA database [113]. This introduces taxonomic bias and may reduce the accuracy of microbial composition data. Furthermore, strain-level variation within the same genus is often overlooked, meaning that associations observed at the genus level may not hold true when examined at a strain-level [114, 115]. Finally, 16S sequencing does not allow for accurate functional inference, limiting insights into metabolic pathways and microbial functionality [116]. As such, infant microbiota abundance may not directly reflect functional capacity.

## Conclusion

This study demonstrates that maternal diet during gestation exerts both direct and microbiota-mediated effects on the infant gut microbiota at one month postpartum. Notably, dietary patterns such as MMDS and DII were associated with significant changes in *Veillonella* abundance and infant microbial diversity, effects partially mediated by maternal gut taxa including *Coprococcus*, *Eubacterium*, and *Christensenellaceae R-7 group*. These findings emphasize the ecological complexity of maternal-infant microbial transfer and underscore the importance of upstream saccharolytic fermenters in shaping substrate availability for early colonizers like *Veillonella*. While cross-sectional design and methodological constraints limit causal inference, this study highlights the value of incorporating microbial mediation models to disentangle the pathways by which maternal diet influences early-life microbiota development. Future research should employ metagenomic techniques and adopt longitudinal study designs to validate findings and enhance the understanding of these associations and better inform public health recommendations.

## Supplementary Information

Below is the link to the electronic supplementary material.

Supplementary file1

Supplementary file2

## Data Availability

Data described in the manuscript, code book, and analytic code are publicly and freely available without restriction at Github [https://github.com/EFV1995/CMA_and_PCA_analysis_scripts]. Raw sequences are available through the NCBI Sequence Read Archive Database under project accession number BioProject ID PRJNA614975.

## References

[1] Sarkar A, Yoo JY, Valeria Ozorio Dutra S et al (2021) The association between early-life gut microbiota and long-term health and diseases. J Clin Med. 10.3390/jcm10030459 33504109 10.3390/jcm10030459 PMC7865818

[2] Barker DJP, Godfrey KM, Gluckman PD et al (1993) Fetal nutrition and cardiovascular disease in adult life. Lancet 341:938–941. 10.1016/0140-6736(93)91224-A 8096277 10.1016/0140-6736(93)91224-a

[3] Guo F, Cai D, Li Y et al (2021) How early-life gut microbiota alteration sets trajectories for health and inflammatory bowel disease? Front Nutr. 10.3389/fnut.2021.690073 34422881 10.3389/fnut.2021.690073 PMC8377158

[4] Park H, Koh N-YP (2023) Scarring the early-life microbiome: its potential life-long effects on human health and diseases. BMB Rep 56:469–481. 10.5483/BMBRep.2023-0114 37605613 10.5483/BMBRep.2023-0114 PMC10547969

[5] Liu Y, Jiao C, Zhang T et al (2023) Early-life gut microbiota governs susceptibility to colitis via microbial-derived ether lipids. Research 6:0037. 10.34133/research.0037 37040489 10.34133/research.0037 PMC10076029

[6] Jeong S (2022) Factors influencing development of the infant microbiota: from prenatal period to early infancy. Clin Exp Pediatr 65:438–447. 10.3345/cep.2021.00955 10.3345/cep.2021.00955 PMC9441613 34942687

[7] Grech A, Collins CE, Holmes A et al (2021) Maternal exposures and the infant gut microbiome: a systematic review with meta-analysis. Gut Microbes 13:1–30. 10.1080/19490976.2021.1897210 33978558 10.1080/19490976.2021.1897210 PMC8276657

[8] Valles-Colomer M, Blanco-Míguez A, Manghi P et al (2023) The person-to-person transmission landscape of the gut and oral microbiomes. Nature 614:125–135. 10.1038/s41586-022-05620-1 36653448 10.1038/s41586-022-05620-1 PMC9892008

[9] Feehily C, O’Neill IJ, Walsh CJ et al (2023) Detailed mapping of Bifidobacterium strain transmission from mother to infant via a dual culture-based and metagenomic approach. Nat Commun 14:3015. 10.1038/s41467-023-38694-0 37230981 10.1038/s41467-023-38694-0 PMC10213049

[10] Bogaert D, van Beveren GJ, de Koff EM et al (2023) Mother-to-infant microbiota transmission and infant microbiota development across multiple body sites. Cell Host Microbe 31:447-460.e6. 10.1016/j.chom.2023.01.018 36893737 10.1016/j.chom.2023.01.018

[11] García-Mantrana I, Selma-Royo M, González S et al (2020) Distinct maternal microbiota clusters are associated with diet during pregnancy: impact on neonatal microbiota and infant growth during the first 18 months of life. Gut Microbes 11:962–978. 10.1080/19490976.2020.1730294 32167021 10.1080/19490976.2020.1730294 PMC7524361

[12] Gibson DL, Gill SK, Brown K et al (2015) Maternal exposure to fish oil primes offspring to harbor intestinal pathobionts associated with altered immune cell balance. Gut Microbes 6:24–32. 10.1080/19490976.2014.997610 25559197 10.1080/19490976.2014.997610 PMC4615215

[13] Talsness CE, Penders J, Jansen EHJM et al (2017) Influence of vitamin D on key bacterial taxa in infant microbiota in the KOALA Birth Cohort Study. PLoS ONE 12:e0188011. 10.1371/journal.pone.0188011 29121673 10.1371/journal.pone.0188011 PMC5679631

[14] Cabrera-Rubio R, Pickett-Nairne K, González-Solares S et al (2024) The maternal diet index and offspring microbiota at 1 month of life: insights from the Mediterranean Birth Cohort MAMI. Nutrients. 10.3390/nu16020314 38276552 10.3390/nu16020314 PMC10821217

[15] Selma-Royo M, García-Mantrana I, Calatayud M et al (2021) Maternal diet during pregnancy and intestinal markers are associated with early gut microbiota. Eur J Nutr 60:1429–1442. 10.1007/s00394-020-02337-7 32728880 10.1007/s00394-020-02337-7

[16] Lou YC, Olm MR, Diamond S et al (2021) Infant gut strain persistence is associated with maternal origin, phylogeny, and traits including surface adhesion and iron acquisition. Cell Rep Med 2:100393. 10.1016/j.xcrm.2021.100393 34622230 10.1016/j.xcrm.2021.100393 PMC8484513

[17] Selma-Royo M, Dubois L, Manara S et al (2024) Birthmode and environment-dependent microbiota transmission dynamics are complemented by breastfeeding during the first year. Cell Host Microbe 32:996-1010.e4. 10.1016/j.chom.2024.05.005 38870906 10.1016/j.chom.2024.05.005 PMC11183301

[18] Flores Ventura E, Esteban-Torres M, Gueimonde M et al (2025) Mother-to-infant vertical transmission in early life: a systematic review and proportional meta-analysis of Bifidobacterium strain transmissibility. NPJ Biofilms Microbiomes 11:121. 10.1038/s41522-025-00720-y 40593735 10.1038/s41522-025-00720-y PMC12219069

[19] Laursen MF, Sakanaka M, Von Burg N et al (2021) Bifidobacterium species associated with breastfeeding produce aromatic lactic acids in the infant gut. Nat Microbiol 6:1367–1382. 10.1038/s41564-021-00970-4 34675385 10.1038/s41564-021-00970-4 PMC8556157

[20] Stokholm J, Blaser MJ, Thorsen J et al (2018) Maturation of the gut microbiome and risk of asthma in childhood. Nat Commun 9:141. 10.1038/s41467-017-02573-2 29321519 10.1038/s41467-017-02573-2 PMC5762761

[21] Fujimura KE, Sitarik AR, Havstad S et al (2016) Neonatal gut microbiota associates with childhood multisensitized atopy and T cell differentiation. Nat Med 22:1187–1191. 10.1038/nm.4176 27618652 10.1038/nm.4176 PMC5053876

[22] Fukuda S, Toh H, Hase K et al (2011) Bifidobacteria can protect from enteropathogenic infection through production of acetate. Nature 469:543–547. 10.1038/nature09646 21270894 10.1038/nature09646

[23] Saturio S, Nogacka AM, Alvarado-Jasso GM et al (2021) Role of Bifidobacteria on infant health. Microorganisms 9:2415. 10.3390/microorganisms9122415 34946017 10.3390/microorganisms9122415 PMC8708449

[24] Stuivenberg GA, Burton JP, Bron PA, Reid G (2022) Why are Bifidobacteria important for infants? Microorganisms 10:278. 10.3390/microorganisms10020278 35208736 10.3390/microorganisms10020278 PMC8880231

[25] Seferovic MD, Mohammad M, Pace RM et al (2020) Maternal diet alters human milk oligosaccharide composition with implications for the milk metagenome. Sci Rep 10:22092. 10.1038/s41598-020-79022-6 33328537 10.1038/s41598-020-79022-6 PMC7745035

[26] Yeruva L, Mulakala BK, Rajasundaram D et al (2023) Human milk miRNAs associate to maternal dietary nutrients, milk microbiota, infant gut microbiota and growth. Clin Nutr 42:2528–2539. 10.1016/j.clnu.2023.10.011 37931372 10.1016/j.clnu.2023.10.011

[27] Sindi AS, Stinson LF, Gridneva Z et al (2024) Maternal dietary intervention during lactation impacts the maternal faecal and human milk microbiota. J Appl Microbiol 135:lxae024. 10.1093/jambio/lxae024 38323424 10.1093/jambio/lxae024

[28] Stapleton PD, Shah S, Ehlert K et al (2007) The β-lactam-resistance modifier (−)-epicatechin gallate alters the architecture of the cell wall of *Staphylococcus aureus*. Microbiology 153:2093–2103. 10.1099/mic.0.2007/007807-0 17600054 10.1099/mic.0.2007/007807-0 PMC2063568

[29] Ríos-Covián D, Ruas-Madiedo P, Margolles A et al (2016) Intestinal short chain fatty acids and their link with diet and human health. Front Microbiol 7:185. 10.3389/fmicb.2016.00185 26925050 10.3389/fmicb.2016.00185 PMC4756104

[30] Hales CN, Barker DJ (2001) The thrifty phenotype hypothesis. Br Med Bull 60:5–20. 10.1093/bmb/60.1.5 11809615 10.1093/bmb/60.1.5

[31] Urwin HJ, Miles EA, Noakes PS et al (2014) Effect of salmon consumption during pregnancy on maternal and infant faecal microbiota, secretory IgA and calprotectin. Br J Nutr 111:773–784. 10.1017/S0007114513003097 24128654 10.1017/S0007114513003097

[32] Robertson RC, Kaliannan K, Strain CR et al (2018) Maternal omega-3 fatty acids regulate offspring obesity through persistent modulation of gut microbiota. Microbiome 6:95. 10.1186/s40168-018-0476-6 29793531 10.1186/s40168-018-0476-6 PMC5968592

[33] Savage JH, Lee-Sarwar KA, Sordillo JE et al (2018) Diet during pregnancy and infancy and the infant intestinal microbiome. J Pediatr 203:47-54.e4. 10.1016/j.jpeds.2018.07.066 30173873 10.1016/j.jpeds.2018.07.066 PMC6371799

[34] Chu DM, Antony KM, Ma J et al (2016) The early infant gut microbiome varies in association with a maternal high-fat diet. Genome Med 8:77. 10.1186/s13073-016-0330-z 27503374 10.1186/s13073-016-0330-z PMC4977686

[35] Lundgren SN, Madan JC, Emond JA et al (2018) Maternal diet during pregnancy is related with the infant stool microbiome in a delivery mode-dependent manner. Microbiome 6:109. 10.1186/s40168-018-0490-8 29973274 10.1186/s40168-018-0490-8 PMC6033232

[36] Fan H-Y, Tung Y-T, Yang Y-CSH et al (2021) Maternal vegetable and fruit consumption during pregnancy and its effects on infant gut microbiome. Nutrients. 10.3390/nu13051559 34063157 10.3390/nu13051559 PMC8148194

[37] García-Mantrana I, Alcántara C, Selma-Royo M et al (2019) MAMI: a birth cohort focused on maternal-infant microbiota during early life. BMC Pediatr 19:140. 10.1186/s12887-019-1502-y 31053102 10.1186/s12887-019-1502-y PMC6498642

[38] Lachat C, Hawwash D, Ocké MC et al (2016) Strengthening the reporting of observational studies in epidemiology-nutritional epidemiology (STROBE-nut): an extension of the STROBE statement. PLoS Med 13:e1002036. 10.1371/journal.pmed.1002036 27270749 10.1371/journal.pmed.1002036 PMC4896435

[39] Fernández-Ballart JD, Piñol JL, Zazpe I et al (2010) Relative validity of a semi-quantitative food-frequency questionnaire in an elderly Mediterranean population of Spain. Br J Nutr 103:1808–1816. 10.1017/S0007114509993837 20102675 10.1017/S0007114509993837

[40] Tablas de composición de alimentos del CESNID. In: SEÑ - Sociedad Española de Nutrición. https://www.sennutricion.org/es/2013/05/13/tablas-de-composicin-de-alimentos-del-cesnid. Accessed 8 Jan 2025

[41] Shivappa N, Steck SE, Hurley TG et al (2014) Designing and developing a literature-derived, population-based dietary inflammatory index. Public Health Nutr 17:1689–1696. 10.1017/S1368980013002115 23941862 10.1017/S1368980013002115 PMC3925198

[42] IBM Corp (2023) IBM SPSS Statistics for Windows, version 29.0. IBM Corp, Armonk

[43] Mariscal-Arcas M, Romaguera D, Rivas A et al (2007) Diet quality of young people in southern Spain evaluated by a Mediterranean adaptation of the diet quality index-international (DQI-I). Br J Nutr 98:1267–1273. 10.1017/S0007114507781424 17640424 10.1017/S0007114507781424

[44] Bamia C, Lagiou P, Buckland G et al (2013) Mediterranean diet and colorectal cancer risk: results from a European cohort. Eur J Epidemiol 28:317–328. 10.1007/s10654-013-9795-x 23579425 10.1007/s10654-013-9795-x

[45] Krebs-Smith SM, Pannucci TE, Subar AF et al (2018) Update of the healthy eating index: HEI-2015. J Acad Nutr Diet 118:1591–1602. 10.1016/j.jand.2018.05.021 30146071 10.1016/j.jand.2018.05.021 PMC6719291

[46] Cortes-Macías E, Selma-Royo M, García-Mantrana I et al (2021) Maternal diet shapes the breast milk microbiota composition and diversity: impact of mode of delivery and antibiotic exposure. J Nutr 151:330–340. 10.1093/jn/nxaa310 33188413 10.1093/jn/nxaa310 PMC7850106

[47] Callahan BJ, McMurdie PJ, Rosen MJ et al (2016) DADA2: high resolution sample inference from Illumina amplicon data. Nat Methods 13:581–583. 10.1038/nmeth.3869 27214047 10.1038/nmeth.3869 PMC4927377

[48] Quast C, Pruesse E, Yilmaz P et al (2013) The SILVA ribosomal RNA gene database project: improved data processing and web-based tools. Nucleic Acids Res 41:D590–D596. 10.1093/nar/gks1219 23193283 10.1093/nar/gks1219 PMC3531112

[49] McMurdie PJ, Holmes S (2013) Phyloseq: an R package for reproducible interactive analysis and graphics of microbiome census data. PLoS ONE 8:e61217. 10.1371/journal.pone.0061217 23630581 10.1371/journal.pone.0061217 PMC3632530

[50] Bolar K (2019) STAT: interactive document for working with basic statistical analysis

[51] Wickham H, Chang W, Henry L, et al (2024) ggplot2: create elegant data visualisations using the grammar of graphics

[52] Kassambara A (2023) ggpubr: “ggplot2” Based Publication Ready Plots

[53] Kassambara A, Mundt F (2020) factoextra: extract and visualize the results of multivariate data analyses

[54] Josef Perktold, Skipper Seabold, Kevin Sheppard, et al (2024) statsmodels/statsmodels: Release 0.14.2

[55] Python Release Python 3.12.7. In: Python.org. https://www.python.org/downloads/release/python-3127/. Accessed 30 Apr 2025

[56] Pandas - Python Data Analysis Library. https://pandas.pydata.org/. Accessed 6 May 2025

[57] Joblib: running Python functions as pipeline jobs — joblib 1.5.0 documentation. https://joblib.readthedocs.io/en/stable/. Accessed 6 May 2025

[58] Kronsteiner-Gicevic S, Gaskins AJ, Fung TT et al (2018) Evaluating pre-pregnancy dietary diversity vs. dietary quality scores as predictors of gestational diabetes and hypertensive disorders of pregnancy. PLoS ONE 13:e0195103. 10.1371/journal.pone.0195103 29614105 10.1371/journal.pone.0195103 PMC5882133

[59] Emond JA, Karagas MR, Baker ER, Gilbert-Diamond D (2018) Better diet quality during pregnancy is associated with a reduced likelihood of an infant born small for gestational age: an analysis of the prospective New Hampshire Birth Cohort Study. J Nutr 148:22–30. 10.1093/jn/nxx005 29378041 10.1093/jn/nxx005 PMC6251578

[60] Shapiro ALB, Kaar JL, Crume TL et al (2016) Maternal diet quality in pregnancy and neonatal adiposity: the healthy start study. Int J Obes 40:1056–1062. 10.1038/ijo.2016.79 10.1038/ijo.2016.79 PMC5356926 27133623

[61] Tahir MJ, Haapala JL, Foster LP et al (2019) Higher maternal diet quality during pregnancy and lactation is associated with lower infant weight-for-length, body fat percent, and fat mass in early postnatal life. Nutrients 11:632. 10.3390/nu11030632 30875943 10.3390/nu11030632 PMC6471184

[62] Zhu Y, Hedderson MM, Sridhar S et al (2019) Poor diet quality in pregnancy is associated with increased risk of excess fetal growth: a prospective multi-racial/ethnic cohort study. Int J Epidemiol 48:423–432. 10.1093/ije/dyy285 30590563 10.1093/ije/dyy285 PMC6469312

[63] de Freitas NPA, Carvalho TR, Gonçalves CCRA et al (2022) The dietary inflammatory index as a predictor of pregnancy outcomes: systematic review and meta-analysis. J Reprod Immunol 152:103651. 10.1016/j.jri.2022.103651 35696840 10.1016/j.jri.2022.103651

[64] Laursen MF, Pekmez CT, Larsson MW et al (2021) Maternal milk microbiota and oligosaccharides contribute to the infant gut microbiota assembly. ISME Commun 1:1–13. 10.1038/s43705-021-00021-3 36737495 10.1038/s43705-021-00021-3 PMC9723702

[65] Li W, Tapiainen T, Brinkac L et al (2021) Vertical transmission of gut microbiome and antimicrobial resistance genes in infants exposed to antibiotics at birth. J Infect Dis 224:1236–1246. 10.1093/infdis/jiaa155 32239170 10.1093/infdis/jiaa155 PMC8514186

[66] Rodríguez-Bernal CL, Rebagliato M, Iñiguez C et al (2010) Diet quality in early pregnancy and its effects on fetal growth outcomes: the Infancia y Medio Ambiente (childhood and environment) mother and child cohort study in Spain. Am J Clin Nutr 91:1659–1666. 10.3945/ajcn.2009.28866 20410088 10.3945/ajcn.2009.28866

[67] Shapiro ALB, Kaar JL, Crume TL et al (2016) Maternal diet quality in pregnancy and neonatal adiposity: the healthy start study. Int J Obes (Lond) 40:1056–1062. 10.1038/ijo.2016.79 27133623 10.1038/ijo.2016.79 PMC5356926

[68] Asadi Z, Bahrami A, Zarban A et al (2024) Association of healthy eating index (HEI), alternative healthy eating index (AHEI) with antioxidant capacity of maternal breast milk and infant’s urine: a cross-sectional study. Sci Rep 14:24053. 10.1038/s41598-024-73169-2 39402064 10.1038/s41598-024-73169-2 PMC11473730

[69] Sivula E, Puharinen H, Hantunen S et al (2024) Maternal dietary indexes are not linked to early childhood wheezing or atopic eczema. Pediatr Allergy Immunol 35:e14099. 10.1111/pai.14099 38425169 10.1111/pai.14099

[70] Monthé-Drèze C, Rifas-Shiman SL, Aris IM et al (2021) Maternal diet in pregnancy is associated with differences in child body mass index trajectories from birth to adolescence. Am J Clin Nutr 113(4):895–904. 10.1093/ajcn/nqaa398 33721014 10.1093/ajcn/nqaa398 PMC8023853

[71] Almulla AA, Augustin H, Ahmed LA, Bärebring L (2024) Dietary patterns during pregnancy in relation to maternal dietary intake: the Mutaba’ah Study. PLoS ONE 19:e0312442. 10.1371/journal.pone.0312442 39436896 10.1371/journal.pone.0312442 PMC11495628

[72] García-Mantrana I, Selma-Royo M, González S et al (2020) Distinct maternal microbiota clusters are associated with diet during pregnancy: impact on neonatal microbiota and infant growth during the first 18 months of life. Gut Microbes 11:962. 10.1080/19490976.2020.1730294 32167021 10.1080/19490976.2020.1730294 PMC7524361

[73] Chatzi L, Mendez M, Garcia R et al (2012) Mediterranean diet adherence during pregnancy and fetal growth: INMA (Spain) and RHEA (Greece) mother–child cohort studies. British J Nutr 107(1):135–145. 10.1017/S0007114511002625 10.1017/S0007114511002625 21733314

[74] Hayat PT, Gargari BP, Sarbakhsh P (2024) The association between diet quality index-international and dietary diversity score with preeclampsia: a case–control study. BMC Womens Health 24:193. 10.1186/s12905-024-03023-0 38515180 10.1186/s12905-024-03023-0 PMC10956302

[75] de Dias Duarte Carvalho Souza M, Bueno Ferreira L, dos Santos LC (2024) The dietary inflammatory index is associated with diet quality and nutrient intake during the gestational period. Nutr Res 125:27–35. 10.1016/j.nutres.2024.02.004 38460227 10.1016/j.nutres.2024.02.004

[76] Stewart CJ, Ajami NJ, O’Brien JL et al (2018) Temporal development of the gut microbiome in early childhood from the TEDDY study. Nature 562:583–588. 10.1038/s41586-018-0617-x 30356187 10.1038/s41586-018-0617-x PMC6415775

[77] Arrieta M-C, Stiemsma LT, Dimitriu PA et al (2015) Early infancy microbial and metabolic alterations affect risk of childhood asthma. Sci Transl Med 7:307ra152-307ra152. 10.1126/scitranslmed.aab2271 10.1126/scitranslmed.aab2271 26424567

[78] Zhang S-M, Huang S-L (2023) The commensal anaerobe *Veillonella dispar* reprograms its lactate metabolism and short-chain fatty acid production during the stationary phase. Microbiol Spectr 11:e0355822. 10.1128/spectrum.03558-22 36975840 10.1128/spectrum.03558-22 PMC10100942

[79] Scheiman J, Luber JM, Chavkin TA et al (2019) Meta-omics analysis of elite athletes identifies a performance-enhancing microbe that functions via lactate metabolism. Nat Med 25:1104–1109. 10.1038/s41591-019-0485-4 31235964 10.1038/s41591-019-0485-4 PMC7368972

[80] Louis P, Flint HJ (2017) Formation of propionate and butyrate by the human colonic microbiota. Environ Microbiol 19:29–41. 10.1111/1462-2920.13589 27928878 10.1111/1462-2920.13589

[81] Gerritsen J, Hornung B, Renckens B et al (2017) Genomic and functional analysis of *Romboutsia ilealis* CRIBT reveals adaptation to the small intestine. PeerJ 5:e3698. 10.7717/peerj.3698 28924494 10.7717/peerj.3698 PMC5598433

[82] Vacca M, Celano G, Calabrese FM et al (2020) The controversial role of human gut Lachnospiraceae. Microorganisms 8:573. 10.3390/microorganisms8040573 32326636 10.3390/microorganisms8040573 PMC7232163

[83] Duncan SH, Louis P, Flint HJ (2004) Lactate-utilizing bacteria, isolated from human feces, that produce butyrate as a major fermentation product. Appl Environ Microbiol 70:5810–5817. 10.1128/AEM.70.10.5810-5817.2004 15466518 10.1128/AEM.70.10.5810-5817.2004 PMC522113

[84] Zhang S-M, Hung J-H, Yen TN, Huang S-L (2024) Mutualistic interactions of lactate-producing lactobacilli and lactate-utilizing *Veillonella dispar*: Lactate and glutamate cross-feeding for the enhanced growth and short-chain fatty acid production. Microb Biotechnol 17:e14484. 10.1111/1751-7915.14484 38801349 10.1111/1751-7915.14484 PMC11129673

[85] Abisado RG, Benomar S, Klaus JR et al (2018) Bacterial quorum sensing and microbial community interactions. MBio. 10.1128/mbio.02331-17 29789364 10.1128/mBio.02331-17 PMC5964356

[86] Xiong F, Dai T, Zheng Y et al (2024) Enhanced AHL-mediated quorum sensing accelerates the start-up of biofilm reactors by elevating the fitness of fast-growing bacteria in sludge and biofilm communities. Water Res 257:121697. 10.1016/j.watres.2024.121697 38728787 10.1016/j.watres.2024.121697

[87] Su Y, Xu M-Y, Cui Y et al (2023) Bacterial quorum sensing orchestrates longitudinal interactions to shape microbiota assembly. Microbiome 11:241. 10.1186/s40168-023-01699-4 37926838 10.1186/s40168-023-01699-4 PMC10626739

[88] Rosero JA, Killer J, Sechovcová H et al (2016) Reclassification of *Eubacterium rectale* (Hauduroy et al. 1937) Prévot 1938 in a new genus *Agathobacter* gen. nov. as *Agathobacter rectalis* comb. nov., and description of *Agathobacter ruminis* sp. nov., isolated from the rumen contents of sheep and cows. Int J Syst Evol Microbiol 66:768–773. 10.1099/ijsem.0.000788 26619944 10.1099/ijsem.0.000788

[89] Waters JL, Ley RE (2019) The human gut bacteria Christensenellaceae are widespread, heritable, and associated with health. BMC Biol 17:83. 10.1186/s12915-019-0699-4 31660948 10.1186/s12915-019-0699-4 PMC6819567

[90] Nogal A, Louca P, Zhang X et al (2021) Circulating levels of the short-chain fatty acid acetate mediate the effect of the gut microbiome on visceral fat. Front Microbiol. 10.3389/fmicb.2021.711359 34335546 10.3389/fmicb.2021.711359 PMC8320334

[91] Mukherjee A, Lordan C, Ross RP, Cotter PD (2020) Gut microbes from the phylogenetically diverse genus *Eubacterium* and their various contributions to gut health. Gut Microbes 12:1802866. 10.1080/19490976.2020.1802866 32835590 10.1080/19490976.2020.1802866 PMC7524325

[92] Jiang F, Song P, Wang H et al (2022) Comparative analysis of gut microbial composition and potential functions in captive forest and alpine musk deer. Appl Microbiol Biotechnol 106:1325–1339. 10.1007/s00253-022-11775-8 35037997 10.1007/s00253-022-11775-8 PMC8816758

[93] Verster AJ, Salerno P, Valls R et al (2025) Persistent delay in maturation of the developing gut microbiota in infants with cystic fibrosis. MBio. 10.1128/mbio.03420-24 39945545 10.1128/mbio.03420-24 PMC11898760

[94] Antosca KM, Chernikova DA, Price CE et al (2019) Altered stool microbiota of infants with cystic fibrosis shows a reduction in genera associated with immune programming from birth. J Bacteriol 201:10.1128/jb.00274-19. 10.1128/jb.00274-19 10.1128/JB.00274-19 PMC6657602 31209076

[95] Hayden HS, Eng A, Pope CE et al (2020) Fecal dysbiosis in infants with cystic fibrosis is associated with early linear growth failure. Nat Med 26:215–221. 10.1038/s41591-019-0714-x 31959989 10.1038/s41591-019-0714-x PMC7018602

[96] Moore RE, Townsend SD (2019) Temporal development of the infant gut microbiome. Open Biol 9:190128. 10.1098/rsob.190128 31506017 10.1098/rsob.190128 PMC6769289

[97] Bäckhed F, Roswall J, Peng Y et al (2015) Dynamics and stabilization of the human gut microbiome during the first year of life. Cell Host Microbe 17:690–703. 10.1016/j.chom.2015.04.004 25974306 10.1016/j.chom.2015.04.004

[98] Wu J, Singleton SS, Bhuiyan U et al (2024) Multi-omics approaches to studying gastrointestinal microbiome in the context of precision medicine and machine learning. Front Mol Biosci 10:1337373. 10.3389/fmolb.2023.1337373 38313584 10.3389/fmolb.2023.1337373 PMC10834744

[99] Muller E, Shiryan I, Borenstein E (2024) Multi-omic integration of microbiome data for identifying disease-associated modules. Nat Commun 15:2621. 10.1038/s41467-024-46888-3 38521774 10.1038/s41467-024-46888-3 PMC10960825

[100] Hernández-Cacho A, García-Gavilán JF, Atzeni A et al (2025) Multi-omics approach identifies gut microbiota variations associated with depression. npj Biofilms Microbiomes 11:1–12. 10.1038/s41522-025-00707-9 40295565 10.1038/s41522-025-00707-9 PMC12038053

[101] Savitz DA, Wellenius GA (2023) Can cross-sectional studies contribute to causal inference? It depends. Am J Epidemiol 192:514–516. 10.1093/aje/kwac037 35231933 10.1093/aje/kwac037

[102] Friel C, Leyland AH, Anderson JJ et al (2024) Healthy prenatal dietary pattern and offspring autism. JAMA Netw Open 7:e2422815. 10.1001/jamanetworkopen.2024.22815 39023891 10.1001/jamanetworkopen.2024.22815 PMC11258593

[103] Zhang Y, Hu Y, Talarico R et al (2024) Prenatal exposure to ambient air pollution and cerebral palsy. JAMA Netw Open 7:e2420717. 10.1001/jamanetworkopen.2024.20717 38980674 10.1001/jamanetworkopen.2024.20717 PMC11234239

[104] Weiner S, Wu Y, Kapse K et al (2024) Prenatal maternal psychological distress during the COVID-19 pandemic and newborn brain development. JAMA Netw Open 7:e2417924. 10.1001/jamanetworkopen.2024.17924 38900424 10.1001/jamanetworkopen.2024.17924 PMC11190810

[105] Newbury JB, Heron J, Kirkbride JB et al (2024) Air and noise pollution exposure in early life and mental health from adolescence to young adulthood. JAMA Netw Open 7:e2412169. 10.1001/jamanetworkopen.2024.12169 38805229 10.1001/jamanetworkopen.2024.12169 PMC11134215

[106] Neu AT, Allen EE, Roy K (2021) Defining and quantifying the core microbiome: challenges and prospects. Proc Natl Acad Sci USA 118:e2104429118. 10.1073/pnas.2104429118 34862327 10.1073/pnas.2104429118 PMC8713806

[107] Byeon S, Lee W (2023) An introduction to causal mediation analysis with a comparison of 2 R packages. JPMPH 56:303–311. 10.3961/jpmph.23.189 10.3961/jpmph.23.189 PMC10415648 37551068

[108] Shrout PE (2011) Commentary: mediation analysis, causal process, and cross-sectional data. Multivar Behav Res 46:852–860. 10.1080/00273171.2011.606718 10.1080/00273171.2011.606718 26736049

[109] Su N, Duijster D, van der Heijden GJMG et al (2024) The role of psychological distress in the relationship of financial strain with oral health and dental attendance in Dutch adults: a mediation analysis based on cross-sectional data. Commun Dent Oral Epidemiol. 10.1111/cdoe.12974 10.1111/cdoe.12974 38750647

[110] Illner A-K, Harttig U, Tognon G et al (2011) Feasibility of innovative dietary assessment in epidemiological studies using the approach of combining different assessment instruments. Public Health Nutr 14:1055–1063. 10.1017/S1368980010003587 21385523 10.1017/S1368980010003587

[111] Carroll RJ, Midthune D, Subar AF et al (2012) Taking advantage of the strengths of 2 different dietary assessment instruments to improve intake estimates for nutritional epidemiology. Am J Epidemiol 175:340–347. 10.1093/aje/kwr317 22273536 10.1093/aje/kwr317 PMC3271815

[112] Naska A, Lagiou A, Lagiou P (2017) Dietary assessment methods in epidemiological research: current state of the art and future prospects. F1000Res 6:926. 10.12688/f1000research.10703.1 28690835 10.12688/f1000research.10703.1 PMC5482335

[113] Park S-C, Won S (2018) Evaluation of 16S rRNA databases for taxonomic assignments using a mock community. Genomics Inform. 10.5808/GI.2018.16.4.e24 30602085 10.5808/GI.2018.16.4.e24 PMC6440677

[114] Yassour M, Jason E, Hogstrom LJ et al (2018) Strain-level analysis of mother-to-child bacterial transmission during the first few months of life. Cell Host Microbe 24:146-154.e4. 10.1016/j.chom.2018.06.007 30001517 10.1016/j.chom.2018.06.007 PMC6091882

[115] Smillie CS, Sauk J, Gevers D et al (2018) Strain tracking reveals the determinants of bacterial engraftment in the human gut following fecal microbiota transplantation. Cell Host Microbe 23:229-240.e5. 10.1016/j.chom.2018.01.003 29447696 10.1016/j.chom.2018.01.003 PMC8318347

[116] Matchado MS, Rühlemann M, Reitmeier S et al (2024) On the limits of 16S rRNA gene-based metagenome prediction and functional profiling. Microb Genom 10:001203. 10.1099/mgen.0.001203 38421266 10.1099/mgen.0.001203 PMC10926695

