# Supplementary material for: Novel insights into how gestational diet affects maternal-infant microbiota: a cross-sectional causal mediation analysis at one month postpartum
Source: Eur J Nutr. 2026 Mar 9;65(3):87. doi: 10.1007/s00394-026-03909-9 (PMC12971879; doi:10.1007/s00394-026-03909-9)
Supplement: Supplementary file 2 — Supplementary file2 [file 394_2026_3909_MOESM2_ESM.docx]

Online Supplementary materials

Novel insights into how gestational diet affects maternal-infant microbiota: a cross-sectional causal mediation analysis at one month postpartum

Eduard Flores Ventura^1^, Sergio Ruiz Saavedra^3,6^, Raul Cabrera-Rubio^1^, Cecilia Martinez-Costa^4,5^, Sonia Gonzalez^2,3^, Maria Carmen Collado^1*^

^1^Institute of Agrochemistry and Food Technology, Spanish National Research Council (IATA-CSIC), Agustin Escardino 7, 46980 Paterna, Spain

^2^Department of Functional Biology, University of Oviedo, 33006 Oviedo, Spain

^3^Diet, Microbiota and Health Group, Instituto de Investigación Sanitaria del Principado de Asturias (ISPA), 33011 Oviedo, Spain

^4^Department of Pediatrics, School of Medicine, University of Valencia, Valencia, Spain.

^5^Pediatric Gastroenterology and Nutrition Section, Hospital Clínico Universitario Valencia, INCLIVA, Valencia, Spain;

^6^Department of Microbiology and Biochemistry of Dairy Products, Instituto de Productos Lácteos de Asturias (IPLA-CSIC), 33300 Villaviciosa, Spain

C. Martinez-Costa, S. Gonzalez, M.C. Collado are equally senior authors

*Corresponding author: MCC, Institute of Agrochemistry and Food Technology- National Research Council (IATA-CSIC), Valencia, Spain, Av. Agustin Escardino 7, 46980 Paterna, Valencia, Spain; Phone +34 96900022; e-mail: mcolam@iata.csic.es

Table 1. Healthy Eating Index-2015 conversion table.

| **Food Group/Subgroup/Nutrient** | **HEI-2015 Component** |
| --- | --- |
| Whole Fruit | Whole Fruit |
| Whole Fruit  Fruit Juice | Total Fruit |
| Whole Grains | Whole Grains |
| Dairy | Dairy |
| Meat, Poultry, Eggs  Seafood  Nuts, Seeds, Soy products.  Legumes | Total Protein Foods |
| Seafood  Nuts, Seeds, Soy products.  Legumes | Seafood and plant proteins |
| Legumes  Dark-Green Vegetables | Greens & Beans |
| Legumes  Dark-Green Vegetables  All Other Vegetables | Total Vegetables |
| Fatty acids | Fatty acids |
| Refined Grains | Refined Grains |
| Sodium | Sodium |
| Added Sugars | Added Sugars |
| Saturated Fats | Saturated Fats |

Table 2. Variables Combined in the Causal Mediation Analysis.

| **Maternal diet indices**  **(Independent variable)** | **Maternal core**  **(Mediator variable)** | **Infant core**  **(Dependent variable)** | **Potential confounders** |
| --- | --- | --- | --- |
| 1. Healthy Eating Index (HEI) 2. Dietary Quality Index (DQI) 3. Dietary Inflammatory Index (DII) 4. Modified Mediterranean Dietary Score (MMDS) | 1. *Adlercreutzia* 2. *Agathobacter* 3. *Akkermansia* 4. *Anaerostipes* 5. *Bacteroides* 6. *Bifidobacterium* 7. *Blautia* 8. *Butyricicoccus* 9. *Christensenellaceae R-7* group 10. *Clostridia UCG-014* 11. *Clostridium sensu stricto 1* 12. *Collinsella* 13. *Coprococcus* 14. *Dorea* 15. *Erysipelatoclostridium* 16. *Erysipelotrichaceae UCG-003* 17. *Eubacterium coprostanoligenes group* 18. *Eubacterium hallii group* 19. *Eubacterium ventriosum* group 20. *Faecalibacterium* 21. *Family XIII AD3011 group* 22. *Fusicatenibacter* 23. *Incertae Sedis* 24. *Intestinibacter* 25. *Monoglobus* 26. *NK4A214 group* 27. *Romboutsia* 28. *Roseburia* 29. *Ruminococcus gauvreauii* group 30. *Ruminococcus* 31. *Ruminococcus torques* group 32. *Streptococcus* 33. *Subdoligranulum* 34. *Turicibacter* 35. *UCG-002* 36. *UCG-005* 37. *Uncultured* 38. Microbial alpha diversity indices: Observed, Chao1, Shannon, and Simpson | 1. *Veillonella* 2. Simpson diversity 3. Shannon diversity | Delivery mode |
